# Origins, Development, and Compartmentation of the Granule Cells of the Cerebellum

**DOI:** 10.3389/fncir.2020.611841

**Published:** 2021-01-15

**Authors:** G. Giacomo Consalez, Daniel Goldowitz, Filippo Casoni, Richard Hawkes

**Affiliations:** ^1^Division of Neuroscience, San Raffaele Scientific Institute, San Raffaele University, Milan, Italy; ^2^Department of Medical Genetics, Centre for Molecular Medicine and Therapeutics, University of British Columbia, Vancouver, BC, Canada; ^3^Department of Cell Biology and Anatomy, Hotchkiss Brain Institute, Cumming School of Medicine, University of Calgary, Calgary, AB, Canada

**Keywords:** granule cell, upper rhombic lip, external granular layer, radial migration, Bergmann glial fibers, compartmentation, cerebellum

## Abstract

Granule cells (GCs) are the most numerous cell type in the cerebellum and indeed, in the brain: at least 99% of all cerebellar neurons are granule cells. In this review article, we first consider the formation of the upper rhombic lip, from which all granule cell precursors arise, and the way by which the upper rhombic lip generates the external granular layer, a secondary germinal epithelium that serves to amplify the upper rhombic lip precursors. Next, we review the mechanisms by which postmitotic granule cells are generated in the external granular layer and migrate radially to settle in the granular layer. In addition, we review the evidence that far from being a homogeneous population, granule cells come in multiple phenotypes with distinct topographical distributions and consider ways in which the heterogeneity of granule cells might arise during development.

## Introduction

In this review article, we address how granule cells (GCs) develop: their origins in the upper rhombic lip (URL: also known as the anterior or rostral rhombic lip) and the elaborate migrations and amplifications that lead GCs to become the most populous neurons in the brain, and secondly the embryological origins of granular layer (GL) heterogeneity. Given the space constraints we do not review synaptogenesis and circuit formation; recent reviews may be found in Leto et al. (2015) and Lackey et al. (2018). This aspect has been covered in several excellent reviews (inter alia Yuzaki, 2011) and in recent research articles that suggest there is much to find out about this critical process (Toledo et al., 2019; Yang et al., 2019; Table 1).

**Table 1:** Summary of genes cited in this review that are involved in granule cell development.

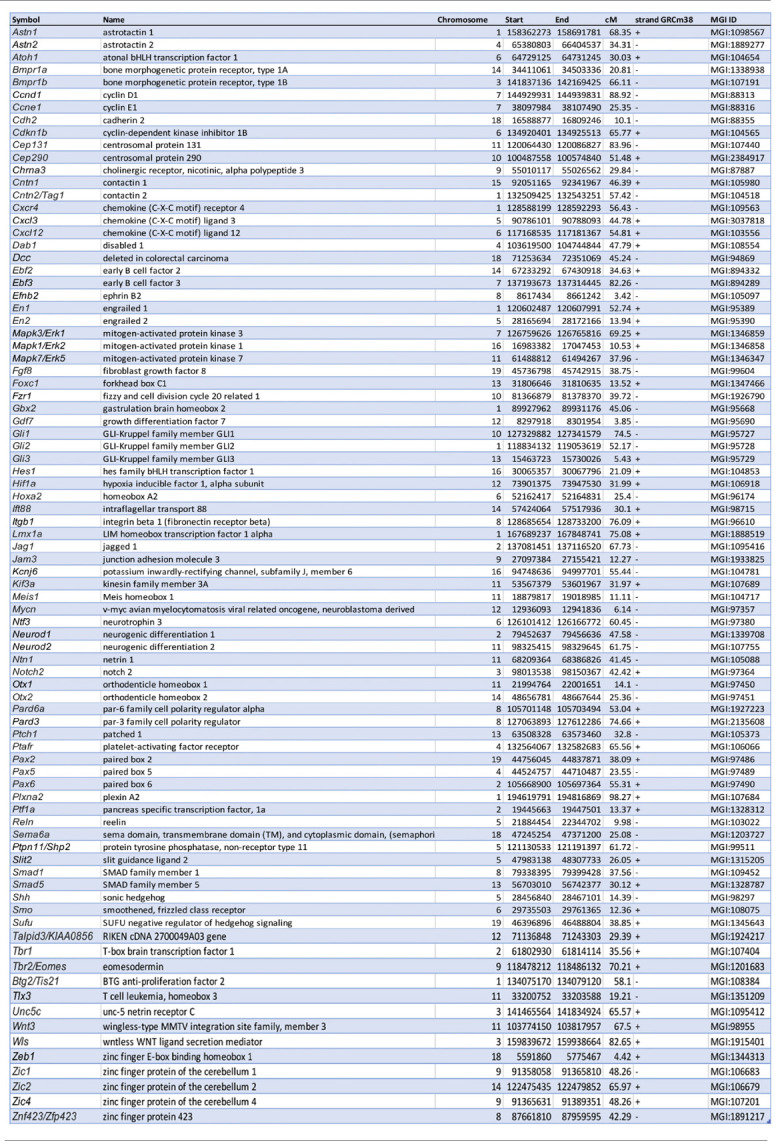

*The “Symbol” and “Name” columns list the symbols and full names of genes, respectively. The “Chromosome” column lists on which chromosome each gene is located. The “Start” and “End” columns list in base pairs along a chromosome at which a gene begins and ends, respectively. The “cM” column gives the length of each gene in centiMorgans (cM). The “strand GRCm38” column reveals whether a gene is located on the positive or negative strand. Finally, the “MGI ID” column provides the ID of each gene on the JAX Mouse Genome Informatics (MGI) website (http://www.informatics.jax.org/). To find out more about cerebellar-specific phenotypes when a gene is perturbed, first copy the MGI ID into the JAX MGI website. Then, on the Gene Summary page, under the “Mutations, Alleles, and Phenotypes” tab, click on the shaded “Nervous System” box. Under the “Mouse Phenotypes” heading, a list of nervous system-related phenotypes is provided, including those that are specific to the cerebellum*.

### A Brief Overview of the Adult Cerebellar Cortex

**Figure 1:**
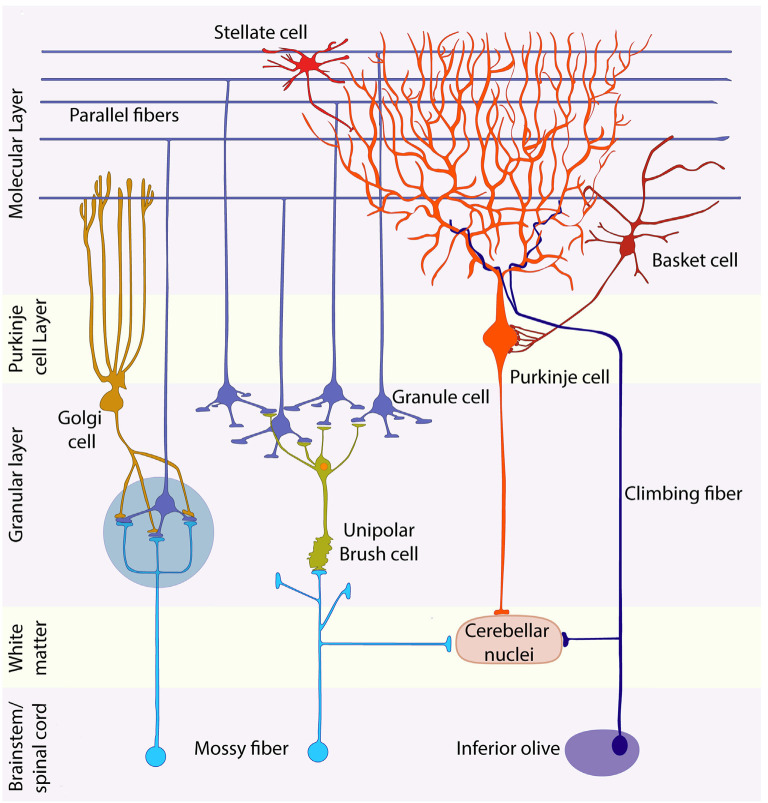
Schematic representation of the cerebellar cytoarchitecture. The cerebellar cortex is organized in three laminae: molecular layer, Purkinje cell (PC) layer, and granular layer (GL). Purkinje, Golgi, stellate, and basket cells are inhibitory neurons; granule and unipolar brush cells are excitatory. Granule cells project their axons into the molecular layer, giving rise to the parallel fibers, which synapse with the dendrites of all inhibitory neurons of the molecular layer and with those of Golgi cells. Afferents are of two main types: mossy and climbing fibers. Both are excitatory. The former originate from brainstem nuclei and the spinal cord, whereas climbing fibers come from the contralateral inferior olive. Most mossy fibers synapse directly on the dendrites of four to five granule cells in specialized synaptic glomeruli (blue-gray circle), which also receive inhibitory feedback from Golgi cell axons. A small mossy fiber subset, first synapses on unipolar brush cells that then relay amplified excitatory signals to granule cells. Each PC receives a connection from a single climbing fiber. Purkinje cell axons carry inhibitory signals to the cerebellar nuclei, whereas mossy and climbing fiber collaterals provide the cerebellar nuclei with excitatory afferents. The large glutamatergic neurons of the CN project their axons to nuclei located in the brainstem and diencephalon.

The adult cerebellar cortex has four distinct layers. Superficially lies the molecular layer, a cell-poor structure rich in GC axons, Purkinje cell (PC) dendrites, and their synapses. Next is the narrow PC layer comprising a monolayer of PC somata. Beneath the PC layer is the GL, densely packed with GC somata. Each of these three cellular layers also has distinct populations of inhibitory interneurons. The three cellular layers overlie the white matter tracts, which comprise the cortical afferent and efferent axon pathways (sketched in Figure 1). Within these layers, all neurons and some glial cells show restriction to a common cerebellar map (e.g., reviewed in Apps and Hawkes, 2009; Apps et al., 2018). Traditionally, the cerebellum has been subdivided into lobes (e.g., anterior, posterior, and flocculonodular; Figure 1), but generally deemed extremely regular (e.g., Marr, 1969). However, on closer inspection, a highly stereotyped pattern of transverse boundaries and parasagittal stripes is revealed, in particular in the distributions of PC phenotypes and afferent terminal fields (recently reviewed in Apps and Hawkes, 2009; Cerminara et al., 2015; Apps et al., 2018). First, the PC layer is comprised of at least five molecularly defined transverse zones—the anterior (AZ), central [CZ: anterior (CZa) and posterior (CZp)], posterior (PZ), and nodular (NZ) zones (Ozol et al., 1999). Each transverse zone is further subdivided into parasagittal stripes, which can be identified by molecular markers (e.g., zebrin II/Aldolase C—Brochu et al., 1990; Ahn et al., 1994); phospholipase Cβ4 (Sarna et al., 2006); heat shock protein 25 (HSP25; Armstrong et al., 2000) and the effects of mutations [rostral cerebellar malformation (rcm) Eisenman and Brothers, 1998; e.g., cerebellar deficient folia (cdf) Beierbach et al., 2001; Niemann Pick disease type C1 (NPC1) Sarna and Hawkes, 2003]. This architecture is reproducible between individuals and conserved across species (e.g., Lannoo et al., 1991; Meek et al., 1992; e.g., reviewed in Marzban et al., 2011). The PC zone-and-stripe array is the ground plan around which other elements are organized. For example, basket and stellate cells (e.g., reviewed in Consalez and Hawkes, 2013), Golgi cells (Sillitoe et al., 2008), unipolar brush cells (UBCs; Chung et al., 2009) and radial glial cells (Eisenman and Hawkes, 1989) all show restriction at PC stripe boundaries, in the sense that either the somata of interneuron subtypes are restricted to particular zones or stripes, or that interneuron neurites do not cross PC stripe boundaries (reviewed in Consalez and Hawkes, 2013). Similarly, afferents are also confined to specific PC stripes (reviewed in Apps et al., 2018) and different stripes exhibit different functional properties (Zhou et al., 2014; Valera et al., 2016). In “Patterning of the Adult Granular Layer” section we describe how this cerebellar compartmentation is reflected in the final arrangement of GCs.

### A Brief Overview of Cerebellar Development

**Figure 2:**
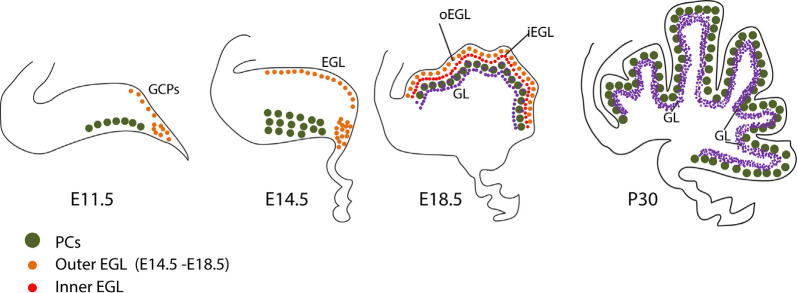
Granule cell development. Schematic representation of granule cell development between E11 and P30. See text for discussion. Abbreviations: EGL, external granular layer; GCPs, granule cell progenitors; GL, granular layer; iEGL, inner lamina of the EGL; oEGL, outer lamina of the EGL; PCs, Purkinje cells; URL, upper rhombic lip.

Cerebellar development (sketched in Figure 2) has been summarized in many reviews (inter alia Leto et al., 2015). The map of the cerebellar anlage is established at early stages [embryonic day (E)8.0–8.5] thanks to extracellular signals released by the isthmic organizer and roof plate and transcription factors expressed rostral and caudal to the isthmus (see Figure 1; Leto et al., 2015). All cerebellar neurons arise from the interplay of two germinal epithelia: the ventricular zone (VZ) and the URL. From ~E9 a small patch in the wall of the VZ of the 4th ventricle (Figure 2), identified by *Ptf1a* expression, is specified to generate all GABAergic neurons—the PCs and multiple classes of inhibitory interneurons (Hoshino et al., 2005; unless otherwise noted all timings refer to mice, with the beginning of embryogenesis designated as E0). The postmitotic PCs migrate dorsally *via* the cerebellar plate (E10–E13) to form physically separate and molecularly distinct clusters by ~E18 (reviewed in Dastjerdi et al., 2012). This stereotyped array is the scaffold around which cerebellar architecture is patterned. The inhibitory interneuron precursors, also derived from the 4th ventricle, migrate *via* the white matter, undergoing further cell divisions *en route*, and then settle in association with the PC clusters (~E18: Leto and Rossi, 2012). Perinatally, the PC clusters and associated interneurons disperse longitudinally to form a monolayer of parasagittal stripes by ~P20 (reviewed in Leto et al., 2015).

**Figure 3:**
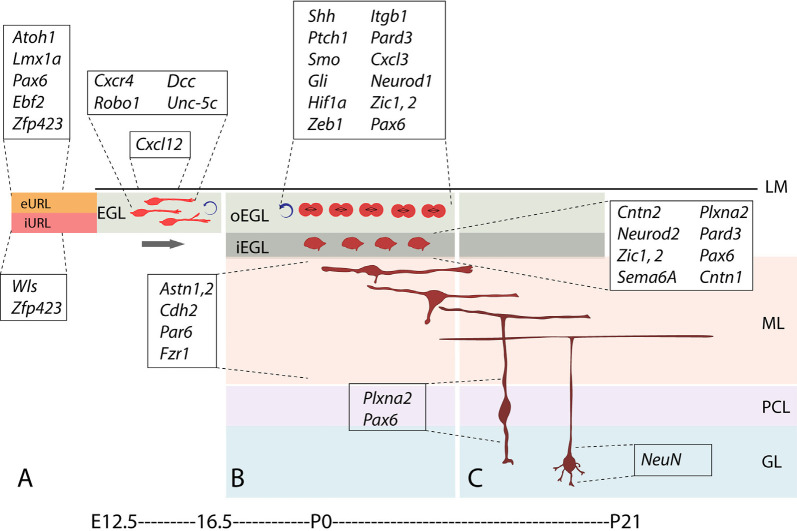
Stages and stage-specific genes of granule cell development. Schematic representation of some genes playing roles in granule cell birth, proliferation, differentiation, and migration. **(A)** *Atoh1+* granule cell progenitors of the external upper rhombic lip (eURL) derive from ill-defined stem cells of the interior URL (iURL) and migrate to give rise to the EGL thanks to a combination of attractive and repulsive cues (see also Figure 4C), and expand in number between E12.5 and E16.5. **(B)** After populating the oEGL, GCPs start dividing mostly symmetrically in a process called clonal expansion, promoted by Purkinje cell-secreted sonic hedgehog. Postmitotic GCs form the interior EGL (iEGL) and undertake tangential and radial migration (see text) after extending two axons in the frontal plane (prospective parallel fibers). **(C)** As they begin their descent into the molecular layer (ML), GC somata extend a radially oriented axon and dendrite, and once in the granular layer, stack with an inside-out progression such that the early-born GCs occupy deeper locations in the GL and project their axons to deeper locations in the ML. Abbreviations: eURL, exterior upper rhombic lip; iURL, interior upper rhombic lip; EGL, external granular layer; oEGL, outer lamina of the EGL; iEGL, inner lamina of the EGL; LM, leptomeninges; ML, molecular layer; PCL, Purkinje cell layer; GL, granular layer.

In parallel, GC progenitors (GCPs), together with excitatory cerebellar nuclei neuron progenitors and glutamatergic UBC progenitors, are generated dorsally in the upper rhombic lip (Figure 3A), where they proliferate and migrate to form a secondary germinal epithelium, the external granular layer (EGL), that completely covers the embryonic cerebellar surface [also often called the “external germinal layer.” The original term—due to Ramón Y Cajal (1911)—is “granular” (“*couche granulaire externe*”): fortunately, either way, it abbreviates as EGL. The EGL was first described thoroughly by Hess (1858) and then by Obersteiner (1869), and was known for many years as “Obersteiner’s layer”. GCPs proliferate for 3 weeks in the outer EGL (oEGL; Figure 3B) to generate the postmitotic GCs that invade the inner EGL (iEGL) and migrate radially through the molecular layer to eventually settle in the maturing granular layer (GL; Figures 3B,C). As they migrate they leave behind trailing axons that bifurcate and extend mediolaterally as parallel fibers, which synapse with the PC dendritic arbors (see also Figure 2, Leto et al., 2015).

In the adult, GC somata located in the GL send out four to five short dendrites that receive excitatory (glutamatergic) input from incoming mossy fiber afferents (Figure 1), and long axons that extend through the molecular layer, bifurcate giving rise to parallel fibers (Figure 3C) and form excitatory (glutamatergic) synapses on PC dendrites. The circuit is thus: mossy fiber afferent input is relayed *via* GC axons to the PCs, which are the sole efferent projection of the cerebellar cortex (Figure 1). While the mossy fiber ⇛ GC ⇛ PC pathway appears straightforward, it has two remarkable features. Cerebellar GCs are the most numerous neurons in the brain: in mice, there are estimated to be 70,000,000, which constitute well over 99% of all neurons in the cerebellum (Herculano-Houzel et al., 2006)! It is noteworthy that large numbers of GCs are a highly conserved feature of the cerebellar cortex (the GC/PC ratio goes up during evolution: Ito, 1984). Therefore, large numbers matter. It is less clear why such an enormous number of GCs is needed (e.g., the “combinatorial coding” theory of Marr, 1969).

## Origins and Formation of The Rhombic Lip

The GC lineage arises at around E8.75 from the URL, an ephemeral structure located atop the 4th ventricle at the intersection of the roof plate and the cerebellar anlage. A two-step process formats the cerebellum and the future GCs: first, arealization of rhombomere 1 of the dorsal neural tube forms the initial cerebellar territory, which includes the URL, and second signaling from the adjacent roof plate assists in cell specification. Innovative techniques have been applied to the analysis of the origins of the primordium—from the classical chick-quail chimera technique of LeDouarin and colleagues (e.g., Pourquié et al., 1992) to the creative use of transgenic mouse lines. These studies defined rhombomere 1 as the territory that produces the anlage. The application of molecular techniques, whether *in situ* hybridization (Hallonet and Le Douarin, 1993) or mouse knockouts (McMahon and Bradley, 1990; Millet et al., 1996), further refined the URL to an anterior rhombencephalic region bounded by the isthmic organizer (*Otx2+*) rostrally and rhombomere 2 (*Hoxa2+)* caudally. In the arealization of the URL, *Gbx2* plays an important role through the antagonism of *Otx2* expression (Hashimoto and Hibi, 2012). The isthmic organizer resembles a Spemann–Mangold-like inducer in that its secreted signals can induce cerebellar-like structures when transplanted to ectopic sites and if it is eliminated no cerebellum is produced (e.g., FGF8 Chi et al., 2003). Other molecules documented to play a role in specifying this region as future cerebellum include the Paired-box transcription factor genes *Pax2* and *Pax5* (Bouchard et al., 2000), and *En1* and *En2* of the engrailed family (Hanold, 1986; Hanks et al., 1995).

While not contributing to the cells that come to populate the parenchyma of the cerebellum, the roof plate expresses molecules that are key to URL development. Important insights arise from the study of the *Dreher* mutant mouse (*Lmx1adr-J)* whose mutated gene was found to be *Lmx1a* (Millonig et al., 2000). Studies by Chizhikov et al. (2006) led to an appreciation of this extra-cerebellar signaling center. The loss of LMX1A expression from roof plate cells results in both a major loss of GCs (Sekiguchi et al., 1992) and the ablation of the vermis (Millonig et al., 2000; Sillitoe et al., 2014). *Lmx1a* is also expressed in a subset of rhombic lip progenitors that produce GCs predominantly restricted to the cerebellar posterior vermis. In the absence of *Lmx1a*, these cells precociously exit the rhombic lip and overmigrate into the anterior vermis. This overmigration is associated with premature regression of the rhombic lip and posterior vermis hypoplasia in *Lmx1a* null mice (Chizhikov et al., 2010). LMX1A acts downstream to signaling *via* bone morphogenetic protein receptors (BMPRs) and this pathway is likely involved in the production of the crucial progenitor gene *Atoh1* (*Atonal homolog 1*; a.k.a *Math1*, Alder et al., 1999; Krizhanovsky and Ben-Arie, 2006). Genetic destruction of the roof plate by using diphtheria toxin driven by the roof plate specific gene *Gdf7* resulted in the near-total loss of *Atoh1* cells of the URL (Chizhikov et al., 2006). BMPRs assemble into a heterotetramer and phosphorylate members of the SMAD family (Smad1, 5, 8; signaling pathway reviewed in Waite and Eng, 2003). Double knockouts of *Bmpr1a; Bmpr1b* and *Smad1; Smad5* result in a dramatic loss of GCPs that is attended by loss of *Atoh1* and other critical genes in the GC lineage including *Zic1* and *Zic2* (Qin et al., 2006; Tong and Kwan, 2013). Interestingly, BMP signaling has also been implicated in the degradation of ATOH1 (Zhao et al., 2008), an effect promoted by *Meis1* and *Pax6* (Owa et al., 2018).

*Atoh1* (Figure 5A) is currently viewed as the definitive marker of the GC lineage, as well as of the other glutamatergic cells that arise from the URL (Akazawa et al., 1995; Ben-Arie et al., 1996). This opened up the molecular analysis of GC development by using transgenesis for gene knockouts and lineage tracing. Of note, evidence was brought to bear on possible upstream genes to *Atoh1*, for example, *Hes1* (Akazawa et al., 1995). This issue is still relatively unexplored although the downstream targets of ATOH1 have been well characterized. Critical genes in the pathway to a glutamatergic phenotype (GCs, glutamatergic projection neurons of the cerebellar nuclei, and UBCs) include *Pax6*, *Tbr1*, and *Tbr2*. *Atoh1* deletion results in the elimination of the entire population of GCs in addition to related populations that derive from the full rhombic lip (Ben-Arie et al., 1997; Wang et al., 2005). This dramatic loss places *Atoh1* in the headwaters of the GC lineage. The examination of downstream targets of ATOH1 has identified a set of genes that suggest a broad developmental impact of *Atoh1* on GC development (Klisch et al., 2011; Machold et al., 2011).

**Figure 4:**
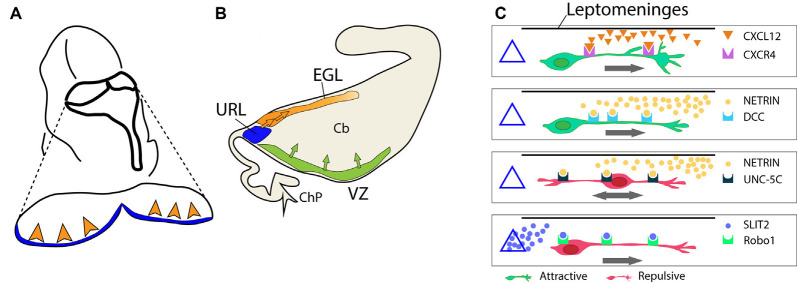
Formation of the external granular layer. **(A)** Schematic representation of the embryonic hindbrain (dorsal view, *circa* E12.5). The view illustrates the departure of GCPs from the URL and the invasion of the EGL (arrowheads). **(B)** The same process is seen in a sagittal view to show the formation of the EGL. **(C)** A sketch of extracellular signals and their receptors controlling GCP migration from the URL into the EGL. The URL is represented as a triangle on the left. The location of the isthmic organizer and mesencephalon is on the right side of each box. CXCL12 is released by the leptomeninges (horizontal black line); SLIT2 is released by the URL (triangle); netrin is secreted by the mesencephalic ventral midline. Abbreviations, URL, upper rhombic lip; EGL, external granular layer; VZ, ventricular zone; ChP, choroid plexus; Cb, cerebellar anlage.

**Figure 5:**
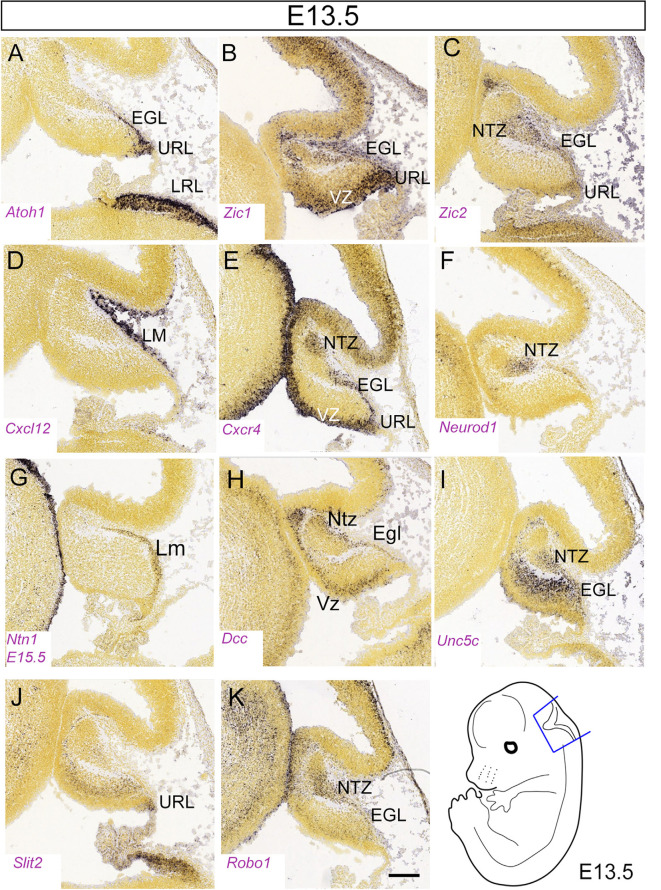
**(A–K) **Distribution of eleven transcripts in the embryonic cerebellar primordium. Sagittal sections hybridized *in situ* with antisense riboprobes specific for genes, cited in the text, that play important roles in the early stages of cerebellar development. Positive territories are labeled black. All images show E13.5 cerebellar primordia, except **(G)**, which shows an E15.5 section. Image credit: Allen Institute. © 2008 Allen Institute for Brain Science. Allen Developing Mouse Brain Atlas. Available online at: https://developingmouse.brain-map.org/. Abbreviations: Cb, cerebellar primordium; ChP, choroid plexus; EGL, external granular layer; NTZ, nuclear transitory zone; URL, upper rhombic lip; VZ, ventricular zone. Scale Bar in **(K)** = 200 μm and applies to all panels.

With the identification of *Atoh1* as key to GC development, it became important to map the timing of the cells that emerge from the *Atoh1* lineage by using site-specific recombinase genetic fate mapping (Dymecki and Tomasiewicz, 1998; Zinyk et al., 1998). The earliest cells to emerge from the URL—between E10.5 and E12.5—are fated to become neurons of the cerebellar nuclei, and give rise to the so-called nuclear transitory zone. They migrate anteriorly over the cerebellar surface as the “rostral rhombic-lip migratory stream” (Wang et al., 2005) or “subpial stream” (Altman and Bayer, 1997; sketched in Figures 4A,B). The GCPs follow the same path from the URL to the EGL. Altman and Bayer’s careful analysis of the rat URL at E10.5 showed two distinct cellular organizations—one tangentially oriented in the exterior lamina of the URL (eURL), and a second with a columnar organization in the interior lamina of the URL (iURL), possibly corresponding to apical radial glial progenitors). We do not know if these laminae are a transitional phase of URL development, but it is clear that they comprise distinct subpopulations based upon the analysis of the *Atoh1* null mouse, where the cells of the eURL are absent while the iURL persists in a normal proliferative state (Jensen et al., 2004). Developmental analysis of the *Wnt* pathway gene *Wntless* (*Wls*; signaling cascade reviewed in Clevers and Nusse, 2012), which processes *Wnt* for its extracellular signaling role, confirms this heterogeneity with the identification of a population of *Wls+* cells in the iURL that are both *Atoh1*-independent [i.e., they persist in the Atoh1 knockout and do not express Atoh1 or the corresponding protein (Yeung et al., 2014)]. In the wildtype, an examination of the two URL laminae suggests that the transition from *Wls*+ to *Atoh1*+ in the iURL serves as a reservoir for the production of ATOH1+ cells and cells of the glutamatergic lineage generally. Genetic fate-mapping of the *Wls*+ population would provide insights into this possibility.

### The URL as the First Zone of Transit Amplification

The expansion of the GCP population during the formation of the EGL from the URL can be thought of as an example of transit amplification—comparable to the transit amplification of GABAergic interneurons as they migrate from the subventricular zone of the 4th ventricle through the white matter tracts (e.g., Leto and Rossi, 2012). In support of this perspective, Wingate and coworkers have shown GCP transit amplification in teleosts, from which a well-defined EGL is absent (Chaplin et al., 2010). The molecular signals that direct URL progenitor cells to form the EGL include the antagonistic interplay between ATOH1 and LMX1A (Chizhikov et al., 2010). Such a tug-of-war between molecularly distinct compartments—i.e., an interplay between factors that push forward developmental events and those which inhibit that progression—is a common dynamic in CNS development (e.g., Toresson et al., 2000; Yeung et al., 2014; Kullmann et al., 2020). Any quantitative estimate of the initial phase of amplification of the GCP population in the proliferatively-active URL is bound to be uncertain as this population gives rise not only to GCs but also to cerebellar nuclear neurons and UBCs.

### From URL to EGL: The Second Stage of Transit Amplification

**Figure 6:**
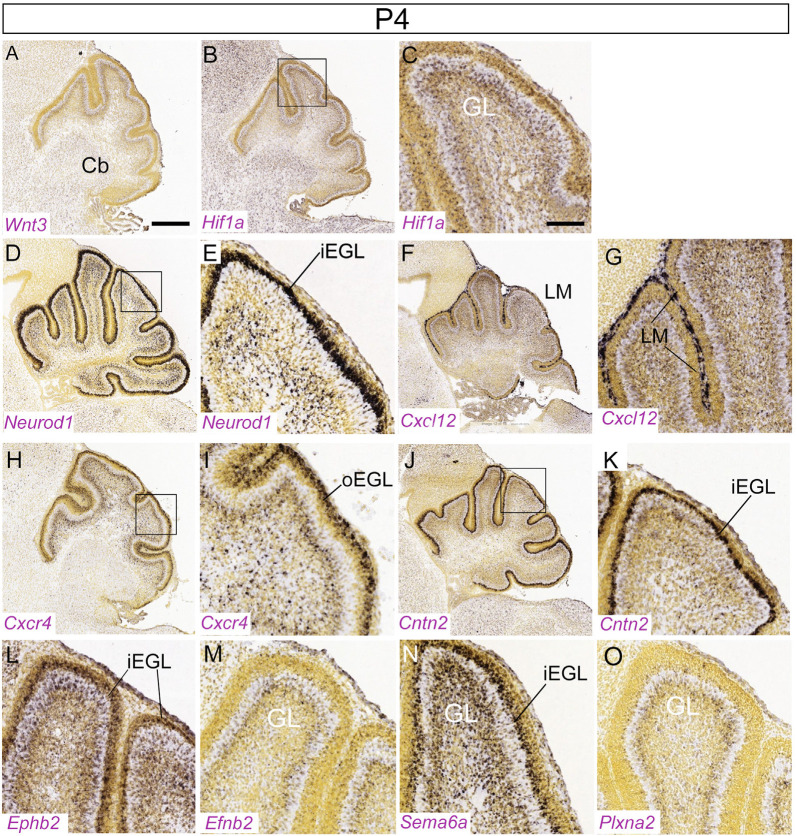
Distribution of nine transcripts in the P4 cerebellum. Sagittal sections hybridized *in situ* with antisense riboprobes specific for genes cited in the text that play important roles at early stages of cerebellar development. Image credit: Allen Institute. © 2008 Allen Institute for Brain Science. Allen Developing Mouse Brain Atlas. Available online at: https://developingmouse.brain-map.org/. Abbreviations: Cb, cerebellum; iEGL, inner EGL; oEGL, outer EGL. **(C,E,J)** are magnifications of areas in panels **(B,D,I)**, respectively. Scale bar in panel (**A**; 400 μm) applies to **(B,D,F,H,J)**; scale bar in panel (**C**; 100 μm) applies to **(E,G,I,K–O)**.

Once GCPs exit the URL to form the EGL, starting at E13, we estimate that from the time that the EGL covers the cerebellum (~E15) to the adult population in the GL (~P25), there is a ~3,000× amplification! As *Atoh1*+ cells exit the URL, they proliferate and disperse tangentially to cover the entire dorsal surface of the cerebellum as the EGL (Figure 3B). Scant information is available about the molecules that guide GCP migration at this stage. One key factor is chemotactic stromal cell-derived factor 1, encoded by the *Cxcl12* gene (Figures 4C, 5D, 6F,G) expressed by the developing leptomeninges and its receptor, CXCR4 (Figures 4C, 5E, 6H,I), expressed by migrating progenitors of the EGL and acting through its downstream effector Shp2 (Hagihara et al., 2009). Fetal cerebellar development in *Cxcl12* mutant animals is markedly different from that in wild-type animals, with many proliferating GCs invading the cerebellar anlage (Zou et al., 1998). Mutations in *Cxcl12* and *Cxcr4* have the same effect on GCP migration, pointing to a monogamous relationship between the corresponding proteins: in the mutant, GCPs depart prematurely from the EGL migrating radially and forming large cell clumps in the cerebellar parenchyma (Ma et al., 1998).

During GCP migration into the EGL (Figures 4A,B; reviewed in Chédotal, 2010), the repulsive extracellular signal SLIT-2 is expressed in the URL (Figure 5J) and probably propels migrating ROBO-expressing GCPs (Figures 4C and 5K) out of the URL (Gilthorpe et al., 2002). GCPs also express the chemoattractant netrin receptor *deleted in colorectal carcinoma* (DCC, Moore et al., 2007). Netrins are a family of laminin-related secreted proteins that direct axon extension and cell migration during neural development. They act as attractants for some cell types and as repellents for others, mediated by distinct receptors (Figure 4C). Among other expression sites, *Ntn1*, encoding netrin-1, is expressed in the mesencephalic ventral midline (Figure 5G). GCPs express the netrin receptor gene *Dcc* (Figure 5H), which mediates the attractive response to netrin-1 during EGL formation. Co-expression of the UNC-5C co-receptor converts the response from attractive to repulsive (Figure 4C). GCP migration is confined within the cerebellar anlage by the netrin co-receptor encoded by *Unc5c* (a.k.a *Unc5h3*; Figure 5I), which acts cell-autonomously to enact a repulsive response to the netrin-1 ligand (Ackerman et al., 1997; reviewed in Goldowitz et al., 2000), thereby preventing the inappropriate anterior migration of GCPs into the inferior colliculus. Consistent with this, in *Pax6^Sey/Sey^* mice, in which *Unc5c* is absent, GCPs are not restricted to the cerebellum and migrate ectopically into the inferior colliculus (Engelkamp et al., 1999). Importantly, however, netrin-1 is not expressed at the anterior limit of the cerebellum and it does not repel GC precursors in collagen-gel assays (Alcantara et al., 2000; Gilthorpe et al., 2002). Furthermore, the EGL forms normally in netrin-1 KO mice (Przyborski et al., 1998; Alcantara et al., 2000), possibly suggesting the presence of redundant signals. Another interesting feature of CGPs that migrate to establish the EGL is that they release reelin (D’Arcangelo, 2014), which guides neuronal migration in the cerebellum as it does in the developing telencephalic cortical plate. In this respect, we view the cerebellar GC as equivalent to the Cajal-Retzius neuron of the isocortex.

### Clonal Expansion in the EGL

Long before the bulk of clonal expansion, DNA synthesis is detected in the prospective EGL. It is not clear whether GCPs divide after completing their migration or combine migration and proliferation simultaneously, similar to basal progenitors of the cerebral cortex. Prenatal migration of GCPs over the cerebellar primordium (E13–E17.5) and subsequent EGL maintenance (E18–P20) both require contacts with the basement membrane, involving both the basal endfeet of radial glial progenitors and fibroblasts of the pia mater. In 1985 Hausmann and Sievers (1985) identified in the E14 rat (roughly corresponding to E12.5 in the mouse) an EGL cell type oriented tangentially to the cerebellar surface and characterized by persistent contact with the basal lamina *via* an external process with a lamellipodial tip and a cytoskeleton characteristic of migratory cells. They proposed that the basal lamina guides the tangentially migrating GCPs and that persistent contacts with the basal lamina mediate stimuli that maintain GCPs in a proliferative state. The bulk of proliferation starts shortly before birth and continues for about 15 days, peaking around P6 (Figure 3B). Clonal expansion requires a mitogenic signal released by neighboring PCs (Smeyne et al., 1995). The nature of this signal is well established—PCs release the morphogen and mitogen sonic hedgehog (SHH) that promotes a massive GCP proliferation (Dahmane and Ruiz-I-Altaba, 1999; Wallace, 1999; Wechsler-Reya and Scott, 1999); accordingly, *Shh* deletion abolishes GCP expansion (Lewis et al., 2004). GCPs are competent to respond to SHH because they express the receptor Patched-1, located near the base of the cell cilium, and the G-protein-coupled transmembrane co-receptor smoothened (SMO; signaling pathway reviewed in Ruiz i Altaba et al., 2002; reviewed in Di Pietro et al., 2017; Lospinoso Severini et al., 2020). SMO activates an inhibitory G protein that in turn activates GLI transcription factors and promotes cell cycle progression. However, the SHH signaling intermediates that regulate GCP proliferation are only just starting to be defined.

## Granule Cell Birth in The EGL

Although SHH is available across the thickness of the molecular layer, GCPs only proliferate when they are exposed to the microenvironment of the EGL. GCPs that remain in the EGL because of impaired migration continue to proliferate: thus, the EGL acts as a mitogenic niche and migration away correlates with the transition from a proliferative to a nonproliferative state (Choi et al., 2005). Regulators of the proliferative environment include *Hif1a* (Figures 6B,C), *Zeb*, and *Itgb1*; conversely, the Pard gene family promotes cell cycle exit and cell migration (see below and Kullmann et al., 2020).

### Symmetric Division of GCPs

Perinatally, GCPs start proliferating rapidly in the EGL and expand clonally over a ~20-day period. Each GCP undergoes predominantly symmetric divisions, generating two GCPs or two neurons (Legué et al., 2015; Nakashima et al., 2015). Cell divisions are oriented to drive growth along the anteroposterior axis. At the same time, the bases of the fissures act as boundaries for GCP dispersion, allowing each lobe/transverse zone to be a distinct developmental unit and ensuring that appropriate GC numbers are partitioned into each folium (Legué et al., 2015).

As mentioned, clonal expansion of GCPs in the mouse EGL is promoted primarily by SHH secreted by PCs (Dahmane and Ruiz-I-Altaba, 1999; Wallace, 1999; Wechsler-Reya and Scott, 1999). This is also the case in humans (Lewis et al., 2004; Aguilar et al., 2012; Haldipur et al., 2012; Lee et al., 2013). Upon SHH binding, the SHH receptor PTCH1 (Marigo et al., 1996) undergoes a conformational change and releases SMO, which enters the cell cilium triggering the activation of the SHH signaling cascade (reviewed in Hu and Song, 2019). While other factors (codon, brother of codon, and growth arrest-specific 1) also regulate the activation of SHH signal transduction, their role in GCP proliferation has not been fully addressed.

The glioma-associated oncogene homolog (GLI) protein family converts SHH signals into gene expression regulation (Ruiz i Altaba, 1999; Corrales et al., 2004). GLI transcription factors are encoded by three genes: *Gli1*, *Gli2*, and *Gli3* (Ruppert et al., 1988). GLI proteins bind to DNA through zinc finger motifs and activate or repress transcription. SHH signaling promotes GLI-1 accumulation in the nucleus and target gene activation (Mimeault and Batra, 2010). GLI-2 and GLI-3 are transcriptional activators and repressors (Wang et al., 2011; Carballo et al., 2018). Among other GLI target genes, *Mycn*, encoding the N-MYC protein, promotes GCP proliferation (Knoepfler et al., 2002; Kenney et al., 2003; Llinás and Negrello, 2015). Besides regulating the cell cycle progression of GCPs, N-MYC promotes a switch from proliferation to differentiation (Knoepfler et al., 2002; Llinás and Negrello, 2015). Ablation of *Gli3* in *Shh* null mice restores *Mycn* expression (Hu et al., 2006). In addition to *Mycn*, GLI factors activate transcription of *Ccnd* and *Ccne* (encoding cyclin D and E, respectively), and thereby promote GCP proliferation (Hatton et al., 2006).

The notch-2 pathway also promotes the expansion of GCPs. Treatment of GCPs with jagged-1, a Notch-2 ligand, stimulates GCP proliferation, and inhibits GC differentiation (Solecki et al., 2001). Activated notch-2 antagonizes BMP signaling (signaling pathway reviewed in Liu and Niswander, 2005; Zhao et al., 2008) and upregulates *Atoh1* expression (Machold et al., 2007). In neural crest development, WNT signaling negatively regulates SHH and controls cell proliferation and differentiation postnatally (Jacob and Briscoe, 2003; Borday et al., 2012): in GCP development, the WNT-3 ligand may antagonize SHH by post-translationally regulating GLI-3 (Anne et al., 2013). WNT-3 is co-expressed with SHH in the postnatal PC layer (Figure 6A) where it suppresses GCP growth through a non-canonical Wnt signaling pathway that activates prototypic mitogen-activated protein kinases (MAPK)—the Ras-dependent extracellular-signal-regulated kinases 1/2 (ERK1/2) and 5 (ERK5) instead of the canonical β-catenin pathway. Inhibition of MAPK activity by using a MAPK kinase inhibitor reversed the inhibitory effect of WNT3 on GCP proliferation (Aguado et al., 2013).

*Tis21* is an antiproliferative gene expressed in differentiating apical progenitors throughout the neural tube (Iacopetti et al., 1999). Its deletion causes an increased frequency of medulloblastoma. In the developing cerebellum, *Tis21*, expressed in Bergmann glia, downregulates *Cxcl3*, a gene that maintains the proliferative niche acting through its receptor expressed in the EGL and delays radial GCP migration from the EGL. Thus, *Tis21* nullisomy may cause GCPs to remain longer in a mitotic state, making them more prone to transformation (Farioli-Vecchioli et al., 2012).

Finally, oxygen tension is a new and complementary pathway to SHH that regulates GCP proliferation. The vascularization of the EGL is reduced in the early postnatal cerebellum and the region is relatively hypoxic resulting in the cells in the EGL expressing the hypoxia-inducing factor 1a gene (*Hif1a*; Figures 6B,C), which stimulates GCP proliferation. Perturbation of neither the SHH pathway nor the HIF1A pathway impacts the other’s role in driving GCP proliferation (Kullmann et al., 2020). The recent work by Solecki and co-workers identifies a microenvironment in which HIF1A stimulates the expression of the homeobox transcription factor ZEB1, which both enhances restriction to the proliferative niche through *Itgb1* and inhibits the *Pard* family of genes that push cells toward migration (Kullmann et al., 2020). When hypoxia is normalized by vascularization of the EGL, *Hif1a* expression diminishes as does its effect on GCP proliferation.

### Primary Cilia and GCP Proliferation

It is now well established that the transduction of SHH signaling starts in the cell cilium. Millen’s group showed for the first time in 2007 that CNS-specific inactivation of ciliary genes *Ift88* and *Kif3a* causes severe cerebellar hypoplasia and foliation abnormalities, due to a failure of GCP clonal expansion, demonstrating that cilia proteins are essential for normal development and GCP proliferation (Chizhikov et al., 2007; Spassky et al., 2008). As noted earlier, the SMO receptor translocates to the primary cilium in response to SHH, and cilia are required for SMO activity (Corbit et al., 2005; May et al., 2005). Moreover, GLI1–3 and SUFU, a negative regulator of the pathway and interactor of GLI proteins, all reside in the distal tip of the primary cilium. Consistent with these findings, intraflagellar transport proteins and cilia are required for both the transcriptional activator and repressor functions of the GLI proteins, affecting both the proteolytic processing of full-length GLI-3 to the repressor form and the transcriptional activity of GLI-2 (Haycraft et al., 2005; Huangfu and Anderson, 2005; Liu et al., 2005; May et al., 2005). Thus, the primary cilium contains the machinery required for the reception and transduction of SHH signaling, explaining its key role in GCP proliferation. Consistent with this, several ciliopathies affect cerebellar cortical development. Joubert and Meckel’s syndromes are ciliopathies characterized by severe vermis defects, ranging from hypoplasia to aplasia. Joubert syndrome patients exhibit a peculiar brainstem malformation known as the “molar tooth sign.” In 2006, Joubert syndrome patients were identified as carrying mutations in *Cep290* (Valente et al., 2006). The homonymous protein is mostly distributed in proliferating GCs and, at the subcellular level, is concentrated in the centrosome and primary cilium. As mentioned previously, cilia control cerebellar morphogenesis by promoting the expansion of the GCP pool (Chizhikov et al., 2007). GCPs possess a primary cilium with CEP290 at its base. The proliferation and the response to SHH are severely impaired in subjects with Joubert or Meckel syndrome (Aguilar et al., 2012). More recently, *ZNF423/Zfp423*, a gene implicated in rare cases of Joubert syndrome, was found to be required for the response to SHH (Hong and Hamilton, 2016), for DNA repair (Chaki et al., 2012; Casoni et al., 2017) and CGP cell cycle progression (Casoni et al., 2017). Likewise, another gene recently implicated in Joubert syndrome, *KIAA0856*, has been shown to encode TALPID3, a centrosomal protein. Mice carrying a conditional *Talpid3* deletion lack primary cilia and show a thinned EGL, presumably due to reduced GCP proliferation (Bashford and Subramanian, 2019). Taken together, Joubert syndrome GCPs exhibit defective or absent primary cilia and have a low proliferative rate throughout the cerebellar cortex.

ATOH1 has been long known to positively regulate the SHH transduction pathway but is also required for the maintenance of primary cilia, which keep GCPs competent to respond to SHH (as mentioned, the loss of primary cilia causes GCPs to exit their proliferative state). ATOH1 activates ciliogenesis by transcriptionally regulating *Cep131*, whose gene product facilitates the clustering of centriolar satellites at the basal body. Importantly, ectopic expression of *Cep131* counteracts the effects of *Atoh1* loss in GCPs by restoring the proper localization of centriolar satellites and consequently ciliogenesis. This pro-proliferative pathway is also conserved in SHH-type medulloblastoma, a pediatric brain tumor arising from GCPs (Chang et al., 2019).

### GCP Cell Cycle Exit and the Onset of Differentiation

The basic helix-loop-helix transcription factor gene *Neurod1* (Figures 5F, 6D,E) is required for differentiation of GCs, and its absence results in GC death (Miyata et al., 1999), particularly in the PZ and NZ (populated by the late-born GC subset—see below: Cho and Tsai, 2006). Experiments on chick embryos have shown that NEUROD1 plays a key role in terminating the proliferation of GCPs by downregulating the expression of *Atoh1*, thereby promoting postmitotic GC migration towards the GL. Premature misexpression of NEUROD1 in chick suppresses transit amplification in the URL migratory stream and hence the formation of the EGL. Furthermore, misexpression of *Neurod1* after the establishment of the EGL triggers radial migration and downregulates *Atoh1* (Butts et al., 2014). The structurally-related protein NEUROD2 promotes the postnatal survival of both GCs and molecular layer interneurons (Pieper et al., 2019).

The *Zic* gene family, which encodes zinc finger transcription factors implicated in neural induction in the early embryo (Nagai et al., 1997; Nakata et al., 1997; reviewed in Aruga and Millen, 2018), play multiple roles in GC development. *Zic1/2* is expressed robustly in the EGL from E12 through birth (Figures 5B,C). Interestingly, *Zic1* is expressed in the deeper part of the EGL and remains expressed in the GL, supporting the notion that *Zic1* and its paralogs play roles both in GCP differentiation and in regulating their proliferation (Aruga et al., 1994, 2002; Gaston-Massuet et al., 2005; Blank et al., 2011; reviewed in Aruga and Millen, 2018). *Zic* mutants feature variable degrees of patterning defects. Although the lamination in *Zic1^−/−^* mice is normal, the pattern of foliation is perturbed (Aruga et al., 1998; Blank et al., 2011). *Zic2* hypomorphs (*Zic2^kd/kd^*) show a medial fusion defect at E16 but no other obvious histological abnormalities (Aruga et al., 2002). Due to cell cycle dysregulation, *Zic1* null mice display hypoplasia of the vermis and hemispheres and reduced GCP proliferation in the EGL. Mechanistically, expression of cyclin D1 (*Ccnd1*) is reduced both in *Zic1*^−/−^ and *Zic1*^+/–^; Zic2^+/kd^ cerebella, and the expression of mitotic inhibitor p27 (*Cdkn1b*), which inhibits GCP proliferation (Miyazawa et al., 2000), is increased in *Zic1^−/−^* cerebella. Moreover, both ZIC1 and ZIC2 act as critical regulators of GC terminal differentiation by affecting chromatin dynamics (Frank et al., 2015). Finally, patients carrying heterozygous deletions encompassing both *Zic1* and *Zic4*, two genes physically linked on chromosome 3q24, develop Dandy-Walker malformation (DWM; Grinberg et al., 2004; Aruga and Millen, 2018). DWM consists of hypoplasia and upward rotation of the cerebellar vermis and cystic dilation of the fourth ventricle. DWM patients have motor deficits including hypotonia and ataxia; about half have mental retardation and some have hydrocephalus. DWM is a heterogeneous disorder and its family recurrence rate does not match expected Mendelian frequencies.

*Cxcl12*, a chemokine expressed by the leptomeninges (Figure 5D), mentioned previously concerning its role in tangential migration, acts as a chemoattractant during GCP migration from the URL into the EGL (Zhu et al., 2004). Also, GCPs, which express the CXCL12 receptor gene *Cxcr4* (Figures 5E, 6G,H), abnormally invade the cerebellar parenchyma in either *Cxcr4* or *Cxcl12* knockouts (Zhu et al., 2004; Vilz et al., 2005). This suggests that CXCL12/CXCR4-mediated attraction plays a major role in maintaining GCPs at the pial surface. The *Src* homology 2-containing protein tyrosine phosphatase is a crucial downstream signal transducer of *Cxcl12* for GC attraction (Hagihara et al., 2009). Interestingly, *Cxcl12* expression by meningeal cells is positively regulated by the FOXC1 transcription factor expressed by leptomeningeal progenitors (Aldinger et al., 2009). Thus, reduced CXCL12 signaling in the EGL may contribute to the cerebellar malformations observed in Dandy-Walker patients bearing *FOXC1* mutations, and to the severe EGL atrophy in *Foxc1* null mice (Aldinger et al., 2009).

Among other factors known to affect the balance of proliferation and differentiation is neurotrophin-3, which promotes differentiation and exit of GCPs from the EGL (Doughty et al., 1998). Likewise, precocious expression of the migration-associated gene *contactin-1* (*Cntn1*) driven by the *Cntn2* promoter delays but does not arrest GCP proliferation in the EGL (Bizzoca et al., 2003). The two contact-related adhesion molecules, TAG1 (CNTN2; gene expression in Figures 6J,K) and F3/contactin (CNTN1) act antagonistically in the response to SHH, promoting GCP expansion and cell cycle exit/differentiation, respectively (Xenaki et al., 2011). Besides adhesion molecules, extracellular matrix glycoproteins also regulate the response to SHH. In particular, laminins and the integrin receptor subunit α6 accumulate in the outermost EGL, where GCP proliferation is greatest. Laminin strongly enhances *in vitro* proliferation of GCPs induced by SHH. Another matrix molecule, vitronectin, and its integrin receptor subunit α(V) are expressed in the inner part of the EGL, where GCPs exit the cell cycle and start to differentiate. Vitronectin promotes phosphorylation of cyclic-AMP responsive element-binding protein (CREB), whose overexpression is sufficient to induce GC differentiation even in the presence of SHH (Pons et al., 2001; also, see above comments regarding Kullmann et al., 2020).

A brief note about the occurrence of normally-occurring cell death in the EGL is appropriate given that such a large expansion of the GCP pool is required to produce the large number of GCs seen in the mature cerebellum. It has been calculated that about 0.12 to 0.37% of EGL cells undergo pyknosis (Smeyne and Goldowitz, 1989). This is a vanishingly small number of cells that leads one to think that the sort of matching mechanisms that drive programmed cell death in other neural structures is not a major sculptor of the final GC number in the adult.

#### Patterning of the EGL

**Figure 7:**
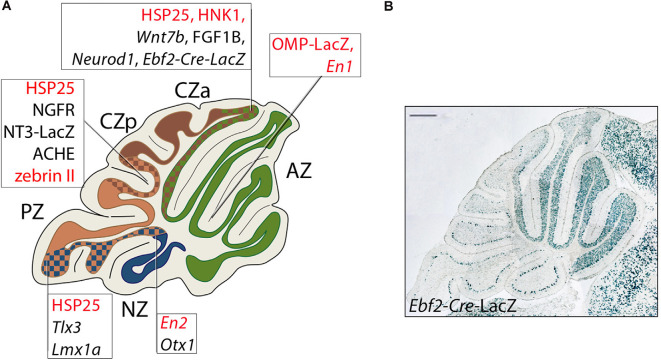
Transverse boundaries established by genes involved in cerebellar development. **(A)** Schematic representation of a midsagittal cerebellar section and its subdivision into PC transverse zones [see text for details: some genes are included that are not expressed in GCs (nor cited in the text) but serve to indicate the locations of transverse boundaries (for a review, see Armstrong and Hawkes, 2013]; PC-specific genes are in red in the text boxes. *Ebf2* is expressed both in GC progenitors and in a PC subset. Lines point to boundaries between adjacent zones identified by the borders of Purkinje cell and granule cell gene expression domains. Abbreviations: AZ, anterior zone; CZa, anterior central zone; CZp, posterior central zone; PZ, posterior zone; NZ, nodular zone. **(B)** An example of the above: a sharp posterior boundary established by GCs derived from *Ebf2+* GCPs populating from URL at E11.5.

Despite its seemingly uniform appearance, the EGL is divided into at least three transverse compartments. For example, transverse boundaries between the AZ and the CZ are seen through the expression of the homeobox genes *Otx1/2* (Frantz et al., 1994), fibroblast growth factor-alpha, and protein tyrosine phosphatase rho (McAndrew et al., 1998), and neurotrophin-3 (Tojo et al., 1995). A similar restriction associated with the PZ/NZ transverse boundary (Figure 7A) is seen for the HOX homeodomain transcription factor gene *Tlx3* (Logan et al., 2002), shown to have a selector role in glutamatergic subtype specification (Shimomura et al., 2015). *Tlx3*, a gene whose expression is induced by PAX6, is expressed in a highly restricted manner in the iEGL and oEGL plus mature cerebellar granule neurons of the posterior cerebellar lobes, where it is required for the localized expression of the nicotinic cholinergic receptor-α3 subunit (CHRN-α3) and other factors involved in neuronal migration and connectivity (Divya et al., 2016). Other transverse boundaries are seen in birth dating studies. The first GCPs are born around E12.5 and preferentially colonize the anterior EGL. Injection of tamoxifen to promote the precisely timed functional activation of *Atoh1*-driven CreER^T2^ at later times (e.g., E16.5–E17) tag GCs that occupy more caudal territories (Machold and Fishell, 2005). The developmental mechanisms by which different GCP populations preferentially occupy particular transverse zones are not certain. In particular, it is not clear whether different GC populations (e.g., anterior vs. posterior) follow different migration pathways or the early-born cells simply migrate further. However, late-born GCPs (*Lmx1a+*) do not move into the anterior cerebellum in mutants in which the early-born (*Lmx1a-*) GCs are absent, again pointing to the presence of an AZ/CZ restriction boundary (see below). Likewise, in the embryonic primordium, the *Ebf2* gene, encoding a helix-loop-helix transcription factor involved in cerebellar cortical development (Croci et al., 2006, 2011; Chung et al., 2008), is expressed in the eURL and the *Atoh1*+ URL migratory stream at E12.5 and downregulated 1 day later. Genetic fate mapping indicates that GCs derived from *Ebf2*+ GCPs end up in the adult AZ (Badaloni et al., 2019), populated by early-born GCs (Machold and Fishell, 2005; Figure 7B). To date it has not been established whether *Ebf2* plays a role in GC development; interestingly, patients carrying mutations of its human paralog *EBF3*, expressed in URL-derivatives of the cerebellar primordium (Croci et al., 2006), exhibit a complex neurodevelopmental syndrome that includes cerebellar ataxia (Sleven et al., 2017).

In like manner, other GCP defects are restricted to the posterior EGL. As hinted above, posterior vermis abnormalities are reported due to mutations in the LIM-homeodomain transcription factor LMX1A, a critical regulator of cell-fate decisions. *Lmx1a* acts downstream of *Atoh1* (Pan et al., 2009) with an expression pattern restricted to a subset of posterior GC progenitors at the border between the URL and the roof plate (Chizhikov et al., 2010). Downregulation of *Lmx1a* is also associated with profound hypoplasia of the choroid plexus (Casoni et al., 2020). These progenitors produce GCs that are predominantly restricted to the posterior EGL and subsequently give rise to the posterior (=NZ; Figure 7A) late-born GCs. Consistent with this, the *Lmx1a* recessive mutant *dreher* (*Lmx1a^dr-J^*) has pronounced GL defects focused on the posterior vermis (Chizhikov et al., 2010). In the absence of *Lmx1a*, mutant CGPs precociously exit the URL and over-migrate into the anterior vermis, leading to a premature regression of the URL and posterior vermis hypoplasia. Finally, the carbohydrate epitope CD15 is transiently expressed in parasagittal stripes in the EGL in both the embryonic human and the developing rabbit cerebellum (Marani and Tetteroo, 1983).

## Postmitotic Granule Cell Radial Migration to The Granular Layer

### Postmitotic GCs Go Through a Wave of Tangential Migration

The translocation of EGL cells to the GL begins with the cells becoming postmitotic and occupying the inner lamina of the EGL, where they form neurites and express the adhesion molecule TAG1, encoded by *Cntn2* (Pickford et al., 1989; Furley et al., 1990; Figure 3B). Once in the iEGL, the somata of the postmitotic GCs migrate tangentially for up to 2 days after their final mitosis. It is worth mentioning that the term “tangential migration” has been legitimately used to indicate mitotically active GCPs plodding along the subpial region, to invade the secondary germinal zone from the URL (e.g., Gilthorpe et al., 2002) and initiate transit amplification and clonal expansion. However, several studies use the term tangential migration to refer to the movement of postmitotic GCs in the iEGL before initiating their radial migration to the GL. During this period, the GCs alter their polarity to emit an axon at either pole: these axons eventually become parallel fibers. This process starts prenatally and continues through ~P21, by which time the EGL is depleted. Tangential migration slows at the interface of the EGL and the growing molecular layer and stops as the GCs extend a single vertical process—the future ascending GC axon—and switch to radial migration (Komuro et al., 2001). The purpose of this tangential migration is unclear: perhaps it serves to align descending GC streams with the gaps (“raphes”) between embryonic PC clusters, thereby ensuring appropriate GC allocation between stripes and zones of the expanding cerebellar cortex (Komuro et al., 2001). Raphes are prominent in chicken (Feirabend, 1990; Lin and Cepko, 1998) but less so in mammals, save for aberrant conditions such as the *Sey* mutant mouse where they are prominent (Swanson and Goldowitz, 2011).

The switch from tangential migration in the EGL to radial migration along Bergmann glial fibers is mediated non-cell-autonomously *via* semaphorin-6A (SEMA6A; Figure 6N) expressed by GCPs (Kerjan et al., 2005) and involves a repulsive interaction with its plexin-A2 receptor (Renaud et al., 2008; gene expression in Figure 6O). Plexin A2 binds to SEMA6A and controls GC migration and nucleus-centrosome coupling cell-autonomously: the lack of SEMA6A only affects tangential migration but not radial migration (Renaud and Chédotal, 2014). In *Sema6A* null mice many GCs remain ectopic in the molecular layer where they go through terminal differentiation and are contacted by mossy fibers. Similarly, expression of the repulsive ephrin receptor EPHB2 (Figure 6L) is restricted to the iEGL. Through reverse signaling, Ephrin B2, encoded by the *Efnb2* gene (Figure 6M) expressed by GCPs of the EGL, inhibits the chemoattractant effect of CXCL12 (Lu et al., 2001), which is expressed in meningeal cells. Finally, as shown by live imaging and functional studies, the SIAH E3 ubiquitin ligase, which controls proteasomal degradation of the PARD3A polarity protein (encoded by the *Pard3* gene), critically regulates GCP adhesion during EGL exit *via* the junctional adhesion molecule JAM-C (Famulski et al., 2010). Additional factors posited to play a role in the switch from tangential to radial migration include: the microtubule-actin crosslinking protein drebrin, interfering with the function of which leads to random movements of both the nucleus and the centrosome and impairs forward motion efficiency (Trivedi et al., 2017); the *Rac* pathway which cell-autonomously controls tangential migration, neurite formation and terminal differentiation of GCPs in the EGL (Nakamura et al., 2017); tenascin (Husmann et al., 1992); tissue plasminogen activator (Seeds et al., 1999); and the platelet-activating factor receptor (Bix and Clark, 1998).

### Radial Migration Is Guided by Bergmann Glial Fibers

The modern study of postmitotic GC migration came from research labs in the Boston area using three novel techniques. First, the rapidly developing field of immunohistochemistry identified glial fibrillary acidic protein and raised antibodies that highlighted radial (Bergmann) glial cells and their Bergmann fibers (Bignami et al., 1972). Second, cell culture analysis offered a means to dynamically study GC migration along Bergmann fibers and thus provide an experimental approach to identify molecules that might play an *in vivo* role in the GC-Bergmann fiber interaction (Wolf, 1970; Hatten and Sidman, 1977; Trenkner and Sidman, 1977; Trenkner et al., 1978). Later, Hatten and Liem showed that in a culture system, glial filaments interacted with cerebellar neurons: the interactions were specific to Bergmann fibers and did not occur with parenchymal astrocytes (Hatten and Liem, 1981). Third, electron microscopy revealed the fine detail of the intimate relations between the migrating GCs and the Bergmann glial fibers (Hatten and Sidman, 1977). As GC somata migrate radially through the nascent molecular layer to the GL, the cell somata extend leading processes along the adjacent radial Bergmann glial fibers (Rakic, 1971).

Live imaging experiments have shown that migrating GCs form an extensive junction beneath the cell surface, mediated by the neuron-glial adhesion protein Astrotactin-1 (ASTN1**)** expressed on migrating GCs (Edmondson et al., 1988; Stitt and Hatten, 1990; Zheng et al., 1996; Adams et al., 2002) and regulates GC migration (Fishell and Hatten, 1991; Zheng et al., 1996). ASTN1 on GCs interacts heterotypically with N-cadherin (CDH2) both in *cis* and in *trans*, to support cell adhesion (Horn et al., 2018), while another family member, Astrotactin-2, regulates ASTN1 trafficking during postmitotic GC locomotion (Wilson et al., 2010). Migrating GCs extend a leading process with short filopodia and lamellipodia that shroud the glial fiber (Edmondson and Hatten, 1987; Gregory et al., 1988). When the neuron-glial adhesion junction beneath the cell body is released the cell soma advances, after which the neuron glides along the glial fiber until a new adhesion forms (Gregory et al., 1988). Migration is coordinated by the PAR-6 polarity complex in the centrosome (Solecki et al., 2004) through a mechanism that includes activation of actomyosin contractile motors in the proximal region of the leading process (Solecki et al., 2009), suggesting that the force needed for the forward movement is provided by contractility in the leading process.

Several ion channels are critical to normal GC radial migration. First is the G-protein coupled, inwardly-rectifying potassium channel GIRK2. A single amino acid mutation in GIRK2 gives rise to the phenotypic picture seen in *weaver* (*Kcnj6^wv^*) mice (Goldowitz and Smeyne, 1995; Patil et al., 1995). Mice carrying the *weaver* mutation (Sidman et al., 1965) exhibit ataxia, mild locomotor hyperactivity, and, occasionally, tonic-clonic seizures. The failure of migration is accompanied by extensive GC death but the extent to which the two are linked is uncertain. GCs in the *weaver* mutant are born normally (Rezai and Yoon, 1972) but subsequently fail to migrate through the molecular layer to the GL owing to faulty interactions with radial glia (Rakic and Sidman, 1973a,b; Bignami and Dahl, 1974; Sotelo and Changeux, 1974; Sotelo, 1975). The abnormal relationship between GCs that had just begun their migration and Bergmann glial fibers led to the hypothesis that the *weaver* gene targeted the glial cell (Rakic and Sidman, 1973c). However, contrary to the initial hypothesis the homozygous *weaver* mutation does not directly affect migration but rather cell-autonomously promotes GC survival and differentiation (Goldowitz and Mullen, 1982; Gao et al., 1992). This is most dramatically shown by the normal migration of wild-type GCs along *weaver* mutant Bergmann glial fibers (Goldowitz and Mullen, 1982). The death of premigratory *Girk2* null GCs in the EGL prompted further studies of the role played by ion channels (Surmeier et al., 1996), particularly calcium currents, in GC migration. Studies conducted by Komuro and Rakic in mouse cerebellar slices showed that selective pharmacological blockade of NMDA-subtype calcium channels impairs cell motility (Komuro and Rakic, 1992) and that the amplitude and frequency components of Ca^2+^ fluctuations correlate positively with the rate of GC movement in cerebellar microexplant cultures (Komuro and Rakic, 1996).

As a result of these processes, postmigratory GCs at the interface of the EGL and molecular layer extend axons several millimeters mediolaterally as parallel fibers, which form excitatory glutamatergic synapses on the Purkinje cell dendrites they intersect. The migrating GC somata express TAG1/CNTN2 and its deletion results in disordered parallel fiber extension (Furley et al., 1990; Dang et al., 2012). The elongation of axons projected by inward-migrating GCs is also disrupted by knockdown of *Fzr1*, encoding an adapter for the anaphase-promoting complex/cyclosome (APC/C) E3 ubiquitin-protein ligase complex (Konishi et al., 2004).

As GCs pass through the molecular layer and settle in the nascent GL they accumulate in an inside-out order (albeit not very strictly so—Legué et al., 2015) such that the earliest born settle adjacent to the white matter tracts, and the late-born cohort ends up more superficially, close to the PC somata. This passive stacking has interesting implications. One is that GCs of different molecular phenotypes (NADPH+/−, Hawkes and Turner, 1994; e.g., dystrophin+/−, Sillitoe et al., 2003) form patches that preferentially occupy either the deep or superficial GL. Thus, a superficial GL patch will project to the more distal PC dendritic arbors while parallel fibers from a deeper patch will terminate on dendritic branches more proximal to the PC somata. It is not clear whether proximal parallel fiber inputs are more influential on PC firing (Gundappa-Sulur et al., 1999; Dorgans et al., 2018). Either way, we believe that molecular differences imply functional differences, and focusing mossy fiber inputs to specific PC dendritic subdomains provides an additional subtlety that may serve to enhance the computational capacity of the mossy fiber pathway.

## Patterning of The Adult Granular Layer

### Granule Cells and Granular Layer Compartmentation

As mentioned, despite its apparent simplicity, it is a mistake to believe that the GL is homogeneous. On the contrary, both expression markers and lineage tracing reveal an intriguing heterogeneity that aligns reliably with the stripe and zone architecture of the PCs, as described briefly in “Introduction” section. For example, consistent lineage restriction boundaries are seen in several models of chimeric mice that express constitutive phenotype markers (sketched in Figure 7A, an example in Figure 7B). Transverse lineage boundaries are often seen in mouse chimeras, and these boundaries align with the location of boundaries between PC transverse zones. For example, in *M. musculus*⟷*M. caroli* chimeras, two GL lineage boundaries are consistently found—one near the AZ/CZ, the other near the PZ/NZ (Goldowitz, 1989): unfortunately, a direct comparison of lineage and PC boundaries has not been made. Furthermore, transverse PC zones interdigitate such that the boundary line is complex. Similar lineage boundaries are seen in embryonic stem cell chimeras (Hawkes et al., 1999). Similarly, in murine chimeras of the *Pax6* mutation *small eye* (*Pax6^Sey/Sey^*) and *ROSA26* controls [*Sey/Sey*⟷*Gt(ROSA)26Sor*], the AZ/CZ boundary is apparent, together with the second boundary in lobules VII/VIII (~CZ/PZ: Swanson and Goldowitz, 2011) such that the *Pax6* mutation preferentially affects the GL of the CZa and CZp. The chimera data are important in that chimeric markers are cell-intrinsic and expression cannot be ascribed to local induction mechanisms. Why do chimera lineages in the GL segregate into discrete transverse zones? One model evokes heterochronicity: autonomous differences in developmental rate between different mouse strains result in one component of the chimera preferentially populating a particular GL compartment (Goldowitz, 1989; e.g., in chimeras the embryonic stem cells develop more quickly and hence end up concentrated in the earlier-born AZ). This view is consistent with the differences in birth dating between GC subtypes.

Many mutations that affect cerebellar development also show GL phenotypes with regional restriction reminiscent of the chimeras, although in these cases it is always a challenge to distinguish intrinsic GC defects from defects in the local environment. For example, in the *meander tail* *(mea/mea)* mutant the anterior cerebellum is agranular with a transition to normal at the AZ/CZ boundary: the phenotype seems intrinsic to GCPs (Hamre and Goldowitz, 1997) with no loss of PCs (Napieralski and Eisenman, 1993). The same phenotype is seen in *rostral cerebellar malformation* [(Unc5crcm): Ackerman et al., 1997; Eisenman and Brothers, 1998] and, at the same location, physical separation of the GL into anterior and posterior parts, with the two overlapping at the AZ/CZ boundary, is seen in the reelin pathway mouse mutant *disabled* (Dab1: Gallagher et al., 1998). Likewise, deletion of the transcriptional activator *NeuroD1* (expression pattern in Figures 5F, 6E) produces a similar agranular phenotype except that GCs are absent posterior to the AZ/CZ boundary (Miyata et al., 1999). Finally, in the heterozygous *weaver* mutant, numerous defects include PC ectopias restricted to the CZ (Eisenman et al., 1998; Armstrong and Hawkes, 2001) and more pertinent here, substantial GC ectopia is seen in the AZ and a transverse GL discontinuity is present in lobules VIII/IX (= PZ/NZ boundary).

Even more striking than the transverse zone and parasagittal stripe boundaries, expression markers in the adult reveal a much more elaborate division of the GL into thousands of small patches (“hyper-heterogeneity”). This was first described for NADPH/nNOS expression (Hawkes and Turner, 1994; Schilling et al., 1994) and subsequently seen also for anti-dysbindin immunostaining (Sillitoe et al., 2003). In the case of NADPH/nNOS, the evidence suggests that heterogeneous expression by GC patches is likely induced by the local mossy fiber environment (Schilling et al., 1994). Evidence of hyper-heterogeneity also comes from an elaborate, reproducible pattern of blebs in the GL that is revealed in paraffin-embedded sections on rehydration (Hawkes et al., 1997, 1998) and by the topography of trigeminal mossy fiber terminal fields (Shambes et al., 1978).

As discussed above, there are intrinsic topographic maps in the PC layer, the EGL, and the GL—all are aligned but how this is achieved is not well understood. The data suggest the following scenario: PC embryonic clusters are the prime organizers and restrict the distribution of different GCP subpopulations in the EGL to align with the underlying transverse PC zones. Thus, as GCPs in the URL proliferate and spread over the surface of the cerebellum to form the EGL (Miale and Sidman, 1961; Smeyne and Goldowitz, 1989) the early-born GCPs (from E12.5)—derived from the specific subset of early-born GCPs that do not express *Lmx1a—*give rise to the anterior EGL (AZ; Figure 7A). Later-born EGL progenitors subsequently migrate to cover the posterior lobe (the CZ and PZ; Figure 7A). The alignment of the PC AZ/CZ boundary with the border between the anterior and posterior EGL compartments—despite their very different embryological origins—suggests that the PC parasagittal architecture restricts GCP dispersal. The EGL transverse boundaries aligned with the CZp/PZ and PZ/NZ presumably arise in similar ways. Perinatally, the EGL boundaries are projected onto the nascent GL as the postmitotic GCs migrate along the radial Bergmann glial fibers and settle. Once the different GC lineages are *in situ*, hyper-heterogeneity appears—as evidenced by expression boundaries, blebs, and trigeminal terminal fields—all reproducible and in register with the overlying PC stripe architecture. It is highly unlikely that GC heterogeneity at this resolution is specified in the URL/EGL so we favor the interpretation that it is secondary to local inductive interactions with PCs and/or mossy fiber afferents (Ozol and Hawkes, 1997). Presumably, GC or PC heterogeneity at the molecular level is a substrate for the fine-tuning of mossy fiber pathways to reflect different input/output requirements and implies the possibility of a much richer mossy fiber input map than is usually appreciated, with up to several thousand parallel afferent pathways (Hawkes and Gravel, 1991).

## Conclusions and Future Developments

While much progress has been made in our understanding of GC development, numerous issues remain unclarified. First, the mechanism through which the *Atoh1*+ URL manages to generate multiple cell types—medial, interpositus, and lateral nuclear neurons, early- and late-born GCs, various UBC subtypes—is not clear. Does *Atoh1* label a population of region-specific stem cells or a heterogeneous pool of committed progenitors? In that case, do these committed progenitors originate from “universal” *Sox2*+ apical progenitors or an, as yet undiscovered, URL-specific, asymmetrically dividing stem cell restricted to glutamatergic fates? And if so, what marks this putative stem cell? An attractive candidate is the *Wls* gene, which labels apical progenitors negative for *Atoh1* (Yeung et al., 2014).

Second, not enough is known about the molecular mechanisms, extracellular cues, and cell-matrix interactions regulating the progression of GCPs from the URL into the EGL. Although previous studies have implicated both chemotactic signals and repulsive cues in the fine regulation of GCP migration into the URL, cell-type-specific inactivation or overexpression approaches may be required to obtain a more complete picture of this process. For example, early-born GCPs (E12.5) populate anterior segments of the EGL (Machold and Fishell, 2005; Badaloni et al., 2019) while later-born ones (E13.5–E15.5) spread throughout the AP axis of the cerebellar primordium, and the latest-born ones (E16.5) are mostly restricted to lobule X (Machold and Fishell, 2005). What molecules regulate their migration at each stage? The raw material to address this question has become available through exciting single-cell analyses of RNA expression over early cerebellar development. These data present a rich resource to mine to identify molecular pathways that would be assignable to various developmental events and epochs (Rosenberg et al., 2018; Hovestadt et al., 2019; Vladoiu et al., 2019; Wizeman et al., 2019).

Third, regarding clonal expansion, recent studies have classified pediatric medulloblastoma (MB) into four distinct molecular subgroups—WNT-dependent, SHH-dependent, Group 3, and Group 4 (reviewed in Northcott et al., 2019). WNT-dependent medulloblastoma has the most benign prognosis, while SHH MB is somewhat more severe (reviewed in Northcott et al., 2019). In particular, Group 3 medulloblastomas often metastasize, resulting in a poor prognosis, while Group 4 metastasizes less frequently and has an intermediate prognosis. Group 4 is the most frequent form of medulloblastoma accounting for 35% of all cases (reviewed in Northcott et al., 2019). Thus, recently discovered signaling pathways underlying clonal expansion (Kullmann et al., 2020) may provide new clues to its diagnosis and management.

Finally, the array of parallel fibers is highly conserved through vertebrate evolution and thus is important, but its development remains poorly understood. The mechanisms that restricts the extension of parallel fibers on the frontal plane (e.g., Berglund et al., 1999), their guidance within and across parasagittal domains, and the selection of their targets in the molecular layer are all largely unexplored. Which mechanisms lead parallel fibers to run orthogonal to the PC dendrites? Is the parallel array due to a cell-intrinsic control of cytoskeletal configuration, as is suggested from the parallel fiber disarray in the *Pax6* mutant *small eye* (*Sey*, Engelkamp et al., 1999; Yamasaki et al., 2001)? Parallel fibers also have implications for cerebellar stripe architecture. Parallel fibers extend several millimeters mediolaterally and intersect, and synapse with, multiple PC stripes. This is curious. Why are mossy fiber terminal fields highly topographically organized and aligned with specific PC stripes (Ji and Hawkes, 1994; Apps et al., 2018) if the parallel fibers promptly throw all the specificity away?

In conclusion, we believe that GC development remains a vital research field, not only because it involves the origin of over half of all neurons in the brain but also for its far-reaching implications in developmental biology, human genetics, oncology, and regenerative medicine.

## Author Contributions

All authors contributed to the article and approved the submitted version.

## Conflict of Interest

The authors declare that the research was conducted in the absence of any commercial or financial relationships that could be construed as a potential conflict of interest.

## Abbreviations

AZ: Anterior Zone
CZa/p: Central Zone, anterior and posterior
EGL: External Granular Layer (oEGL/iEGL: outer and inner laminae)
GC: Granule Cell
GCP: Granule Cell Progenitors
GL: Granular Layer
NZ: Nodular Zone
PC: Purkinje Cell
PZ: Posterior Zone
UBC: Unipolar Brush Cell
URL: Upper Rhombic Lip (eURL/iURL: exterior and interior laminae).
